# Author Correction: *Drosophila* ß_Heavy_-Spectrin is required in polarized ensheathing glia that form a diffusion-barrier around the neuropil

**DOI:** 10.1038/s41467-022-28258-z

**Published:** 2022-02-01

**Authors:** Nicole Pogodalla, Holger Kranenburg, Simone Rey, Silke Rodrigues, Albert Cardona, Christian Klämbt

**Affiliations:** 1 Institut für Neuro- und Verhaltensbiologie, Universität Münster, Münster, Germany; 2 MRC Laboratory of Molecular Biology, Cambridge Biomedical Campus, Cambridge, CB2 OQH, UK; 3 Physiology, Development and Neuroscience Department, University of Cambridge, Cambridge, UK; 4 HHMI Janelia Research Campus, Ashburn, VA USA

**Keywords:** Cellular neuroscience, Blood-brain barrier, Glial biology

Correction to: *Nature Communications* 10.1038/s41467-021-26462-x, published online 4 November 2021.

In this article the affiliation details for Albert Cardona were incorrectly given as Cambridge Biomedical Campus, Cambridge, UK but should have been MRC Laboratory of Molecular Biology, Cambridge Biomedical Campus, Cambridge, CB2 OQH, UK. The original article has been corrected.

