# Supplementary material for: Drosophila ßHeavy-Spectrin is required in polarized ensheathing glia that form a diffusion-barrier around the neuropil
Source: Nat Commun. 2021 Nov 4;12:6357. doi: 10.1038/s41467-021-26462-x (PMC8569210; doi:10.1038/s41467-021-26462-x)
Supplement: Supplementary file 1 — Supplementary Information [file 41467_2021_26462_MOESM1_ESM.pdf]

Supplementary Figures:

Drosophila ß$_{Heavy}$-Spectrin is required in polarized ensheathing glia that form a diffusion-barrier around the neuropil

Nicole Pogodalla[1], Holger Kranenburg[1], Simone Rey[1], Silke Rodrigues[1], Albert Cardona[2,3,4] and Christian Klämbt[1*]

1) Institut für Neuro- und Verhaltensbiologie, Universität Münster, Badestr. 9, 48149 Münster, Germany

2) Cambridge Biomedical Campus, Francis Crick Ave, Cambridge CB2 0QH, United Kingdom

3) Physiology, Development and Neuroscience Department, University of Cambridge, Cambridge CB2 0QH, United Kingdom

4) HHMI Janelia Research Campus, 19700 Helix Dr, Ashburn, VA 20147, USA

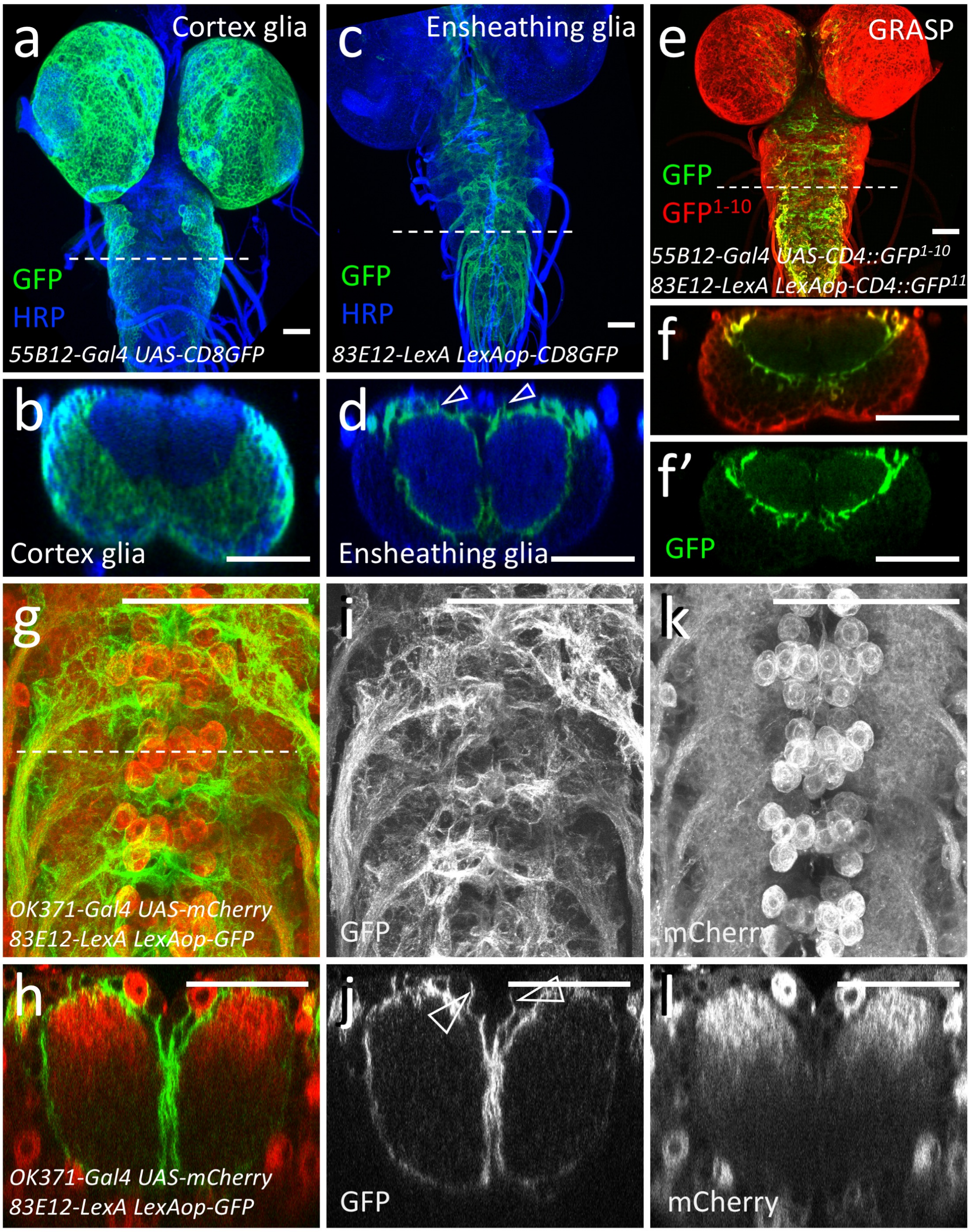

Supplementary Fig. S1 Ensheathing glial cells cover dorsal neurons.

a Frontal view of a ventral nerve cord of a third instar larva. Cortex glial cells are labelled using *55B12-Gal4, UAS-CD8::GFP*, the dashed line shows the position of the orthogonal view shown in (**b**). **b** Orthogonal view, note the absence of cortex glial cell processes dorsally to the neuropil. **c** Frontal view of a ventral nerve cord of a third instar larva with labelled ensheathing glial cells (*83E12-LexA, lexAop-CD8::GFP*), the dashed line shows the position of the orthogonal view shown in (**d**). **d** Orthogonal view, ensheathing glial cell processes cover the entire neuropil. The arrowheads point towards dorsal cell processes engulfing dorsal neurons. **e,f** GRASP experiment. Larvae with the genotype [*55B12-Gal4 UAS-CD4::GFP1-10; 83E12-LexA LexAop-CD4::GFP11*]. Expression of GFP1-10 is detected by an antibody (in red). Reconstituted GFP is shown in green. Note, that no GFP is reconstituted dorsally to the neuropil. **g-l** Ventral nerve cord of a third instar larva with the genotype: [*OK371-Gal4 UAS-mCherry, 83E12-Gal4 UAS-GFP*]. The dashed line indicates the position of the orthogonal section shown in (**h,j,l**). **g-j** The morphology of the ensheathing glial cells is shown by GFP staining (green in **g,h**; white in **i,j**. Glutamatergic neurons are shown in red (**g,h**) or in white (**k,l**). Representative images are shown. Scale bars are 50µm.

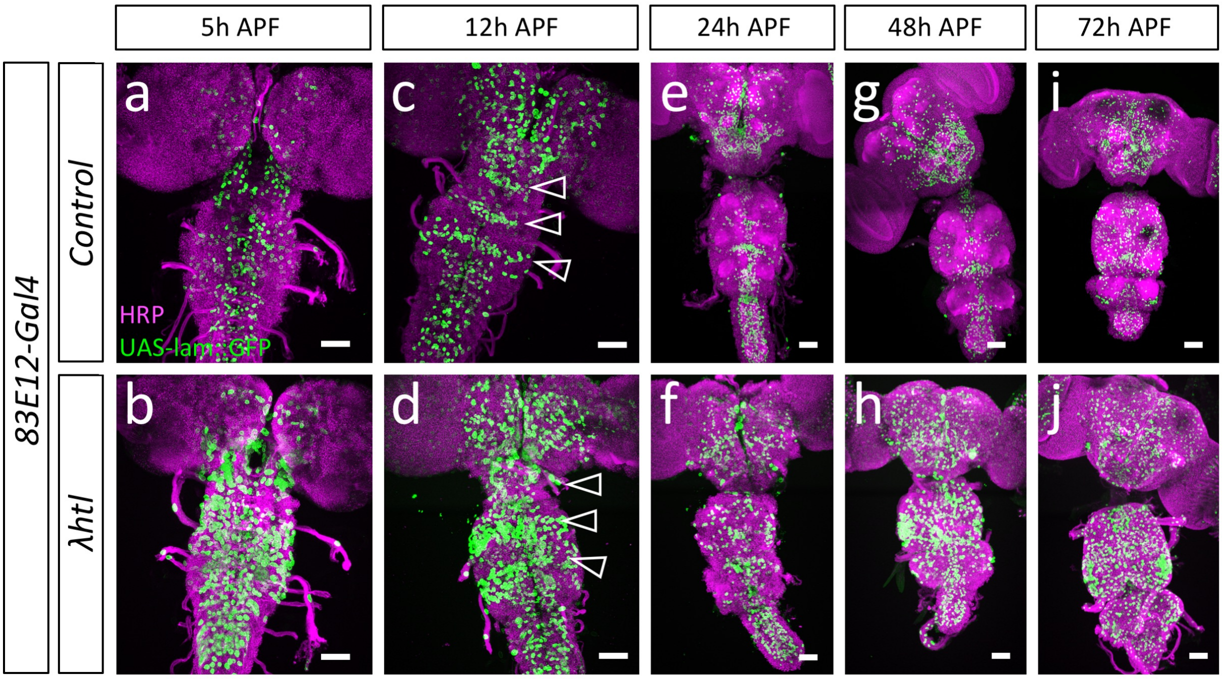

Supplementary Fig. S2 The effect of activated FGF-receptor on ensheathing glia proliferation during pupal stages

Images of pupal brains dissected at the indicated number of hours (h) after puparium formation (APF). Representative images are shown. **a,c,e,g,i** Control animals expressing nuclear GFP in the ensheathing glia [*83E12-Gal4 UAS-Lam::GFP*]. Neuronal cell membranes are shown in magenta (anti-HRP staining), ensheathing glia nuclei are in green (GFP). **b,d,f,h,j** Animals expressing activated FGF-receptor together with nuclear GFP in the ensheathing glia [*UAS-λhlt*, *83E12-Gal4 UAS-Lam::GFP*]. Note the predominant increase of ensheathing glia in the thoracic neuromeres (arrowheads). Scale bars are 50 µm.

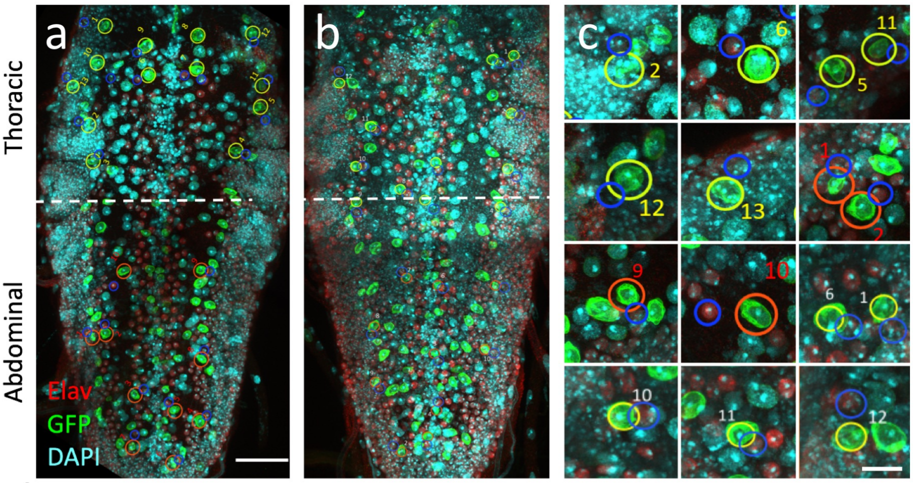

d DAPI ratio

Thoracic Glia/Neuron

Nucleus pair #	1	2	3	4	5	6	7	8	9	10	11	12	13
Brain 1	1,3	1	1	0,8	1	1,9	1,8	1,5	1,8	1,5	1,5	1,3	1,4
Brain 2	1,4	1,4	1,3	1,5	1,2	1,9	1,4	1,4	1,1	1,6	1,6	1,2	
Brain 3	1,7	2,5	1,5	1,7	1	1	1,4	0,9	0,9	2,1			

Abdominal Glia/Neuron

Nucleus pair #	1	2	3	4	5	6	7	8	9	10
Brain 1	2	1,8	1,9	2,3	1,7	2,2	2,4	2	2	1,9
Brain 2	2	2,3	1,7	2,2	1,9	1,9	2	1,7	2,3	2,1
Brain 3	1,9	2,1	2	2,4	3,2	1,8	1,9	1,5	2	2,1

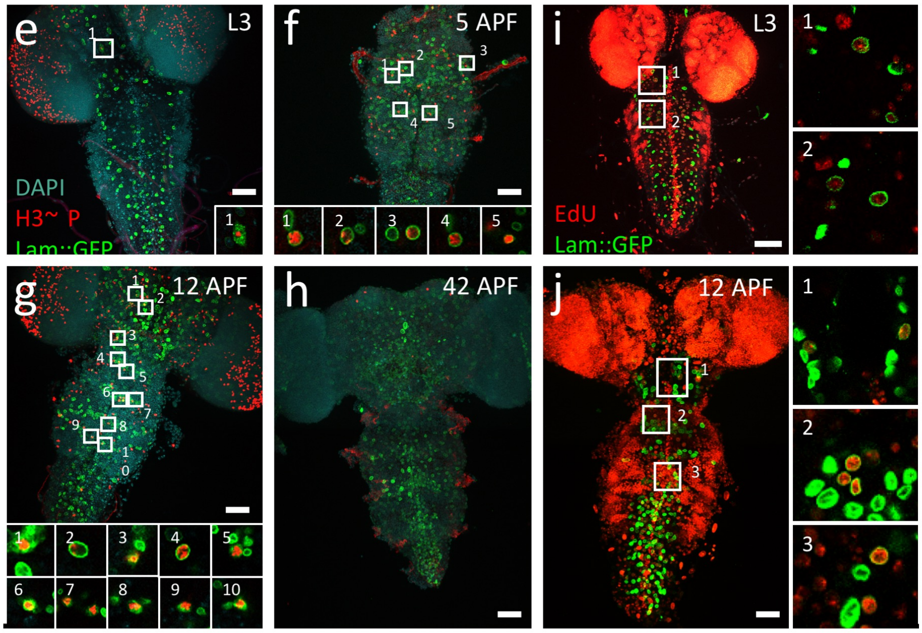

Supplementary Fig. S3 ***83E12-Gal4*** **positive ensheathing glial cell can divide during development**
a,b Maximum projections of two ventral nerve cord stained for neuronal cell nuclei (anti-Elav, red), ensheathing glia nuclei (*83E12-Gal4, UAS-Lam::GFP*, anti-GFP, green) and DAPI (cyan). **c** 12 examples taken from the maximum projection to illustrate neighborhood relationships. Note, that that the analysis of the DAPI signal was conducted all single focal planes with a given nucleus. **d** The ratio of glial and neuronal DAPI intensity is shown for 65 glia / neuron pairs from three brains. Red shading indicates a DAPI intensity ratio of >1.5. **e** Dissected larval CNS with the genotype [*83E12-Gal4, UAS-Lam::GFP*], stained for GFP (green), DAPI (cyan) and phospho-histone H3 (red). **f-h** Dissected pupal CNS of the age indicated with the genotype [*83E12-Gal4, UAS-Lam::GFP*], stained for GFP (green), DAPI (cyan) and phospho-histone H3 (red). Examples of *83E12-Gal4*, phospho-histone H3 positive nuclei are shown in the indicated boxes. **i,j** EdU staining of larval (**i**) and pupal (**j**) brains [*83E12-Gal4; UAS-Lam::GFP*]. EdU is shown in red, Lamin::GFP is shown in green to visualize the nuclei of the ensheathing glia. Examples of *83E12-Gal4*, EdU positive nuclei are shown in the indicated boxes. Representative images are shown. Scale bars are 50 µm, except c: 10 µm. Source data are provided as a Source Data file.

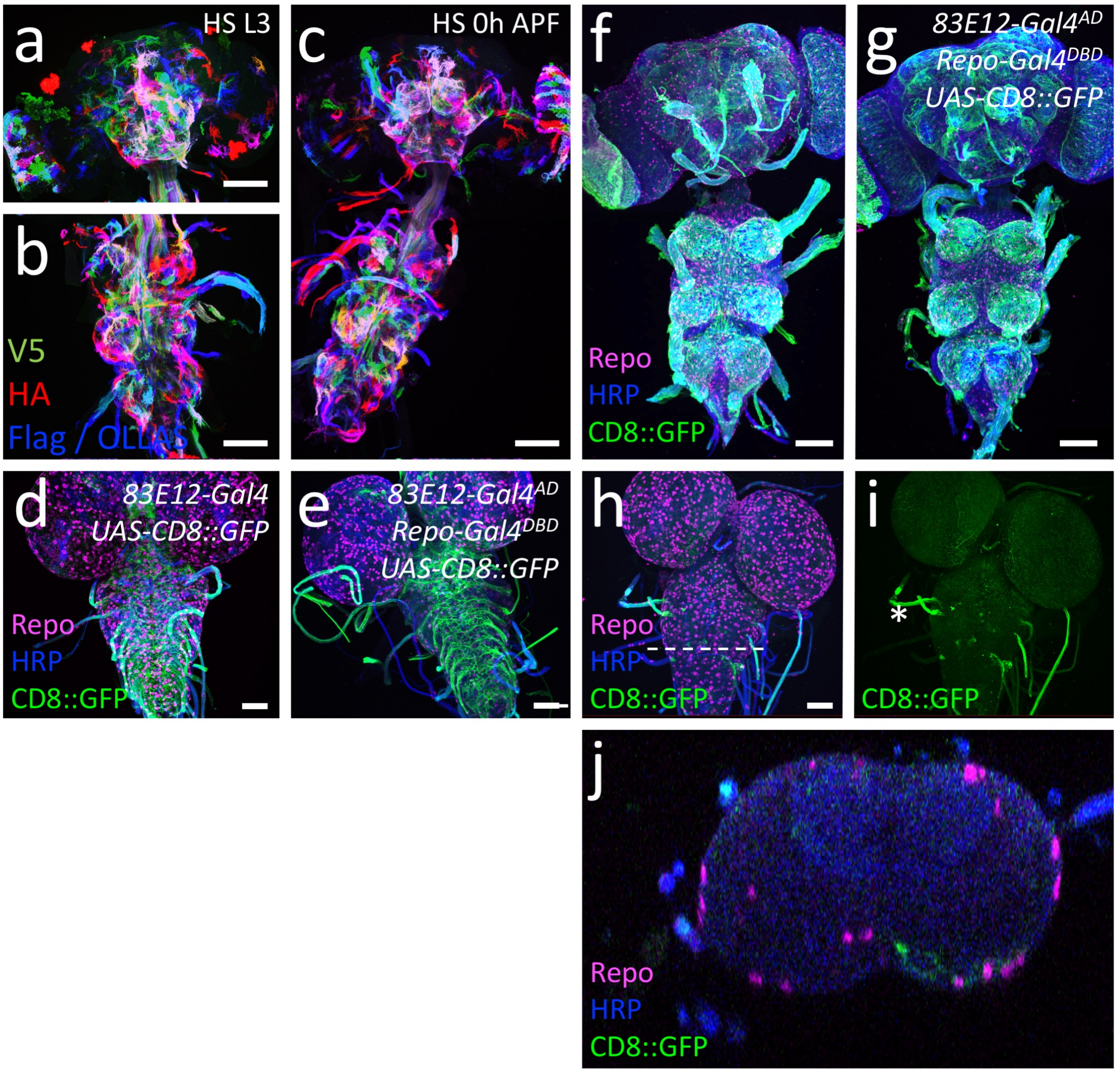

Supplementary Fig. S4 DAPI staining of larval ensheathing glia

a-c MCFO labeling of an adult brain stained for the expression of V5 (green), HA (red), and FLAG and OLLAS epitopes (blue). *flp* expression (HS) was induced for one hour, during third instar larval stage (**a,b**) or at the onset of puparium formation (0h APF, **c**). Note that not all ensheathing glia that are present in the adult CNS are labelled when *flp* is expressed in early development. **d** Third instar larval brain with ensheathing glia labelled using *83E12-Gal4* driving membrane bound GFP. **e** Similar aged larval brain with ensheathing glia labelled using the split-Gal4 combination driving membrane bound GFP [*83E12-Gal4^{AD}*, *repo-Gal4^{DBD}*, UAS-CD8::GFP]. **f,g** Adult brains of animals carrying either the *83E12-Gal4* or the split-Gal4 combination. **h-j** Ablation of ensheathing glia. Animal of the genotype [*83E12-Gal4^{AD}, repo-Gal4^{DBD}, UAS-hid, UAS-CD8::GFP*] lacks detectable GFP-expression in the CNS. Few wrapping glial cells along the peripheral nerve are not ablated (asterisk). The dashed white line indicated the position of the orthogonal section shown in (**j**). Representative images are shown. Scale bars are: larval CNS 50 µm, adult CNS 100 µm.

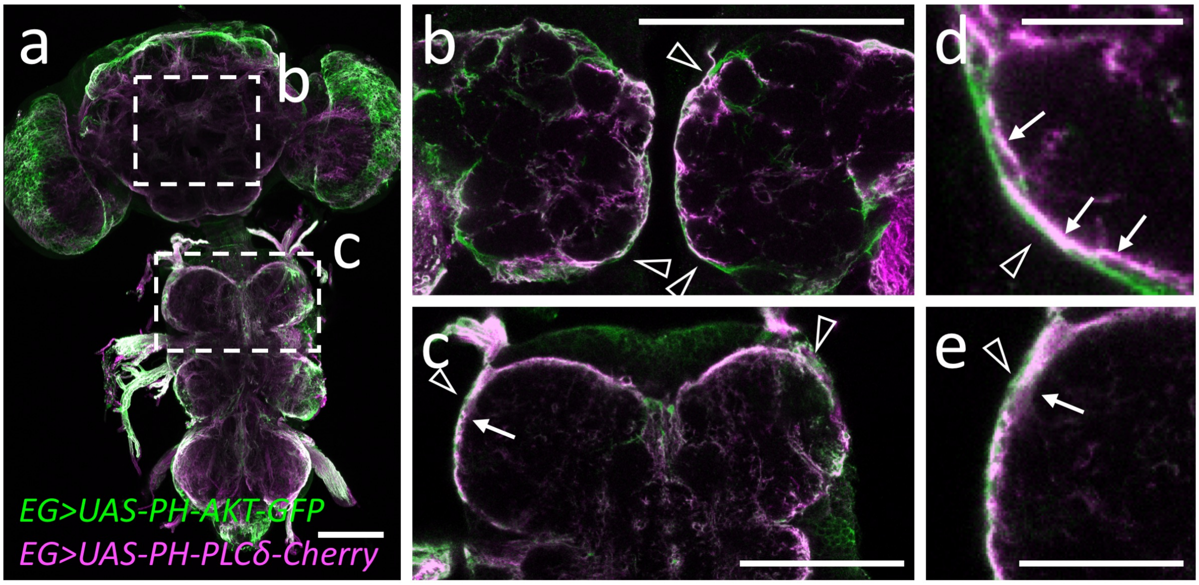

Supplementary Fig. S5 Polar organization of the adult ensheathing glia

a Coexpression of *PH-AKT-GFP* and *PH-PLCδ-mCherry* in adult ensheathing glia (EG). The boxed areas are shown in higher magnification in (**b-e**). Note that green fluorescence indicating PIP_3 is preferentially seen towards cortical regions (arrowheads), whereas magenta staining indicating PIP_2 is preferentially found facing the neuropil (arrows). Representative images are shown. Scale bar is 100 µm except for d,e: 25 µm.

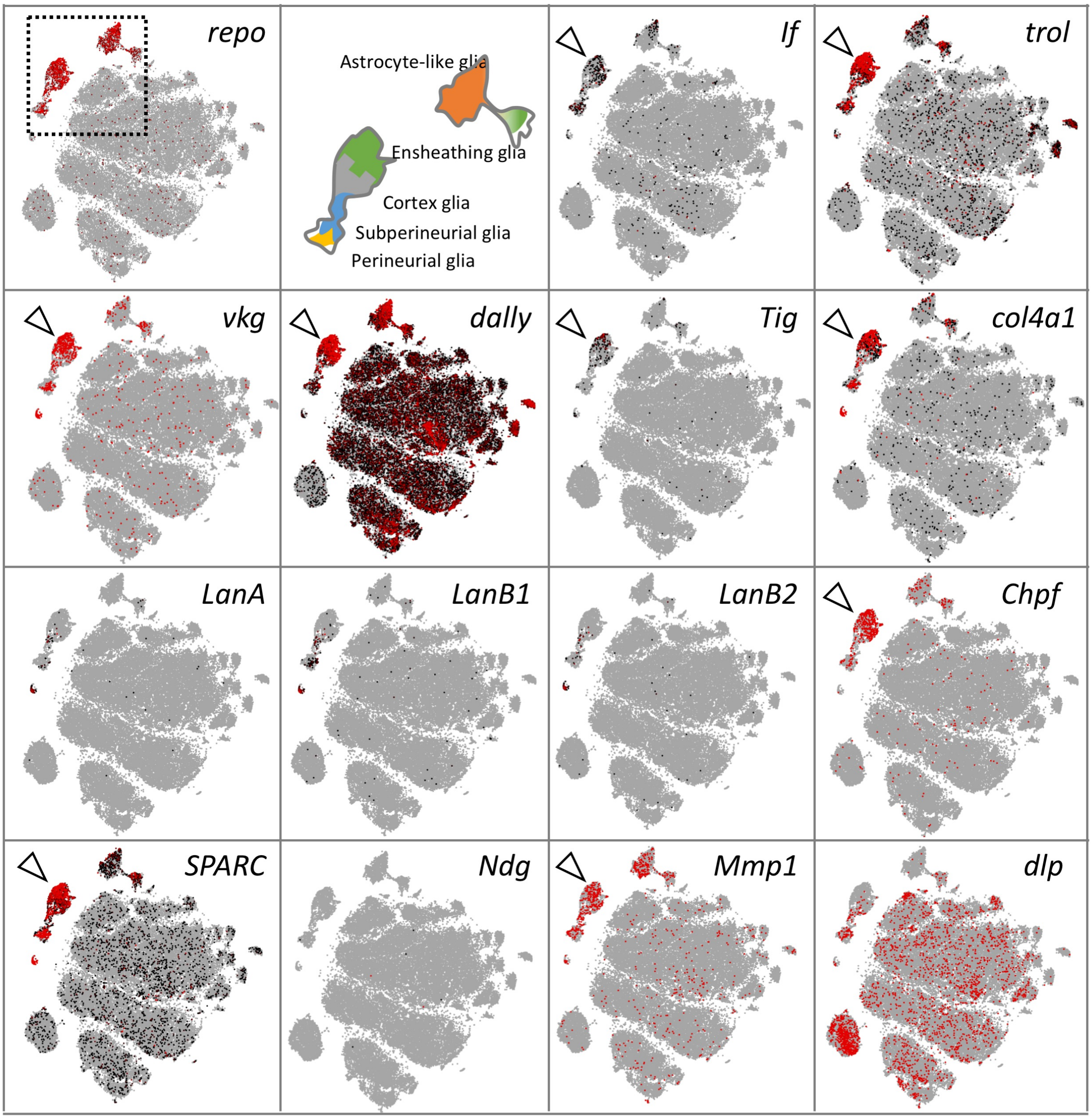

Supplementary Fig. S6 Expression of extracellular matrix components in the adult brain
SCENIC representations of the 57K scRNA seq dataset of the Aerts laboratory [11]. SCope analysis for the genes indicated in each top right corner is shown. Each dot represents a single cell. The color coding indicates the expression level. Red: strong expression, black: low expression, grey: no expression. Expression of the transcription factor Repo defines the glial complement, expression of further markers allows the definition of glial subtypes [11]. *inflated* (*if*) is expressed in ensheathing glia, astrocyte-like glia and perineurial glia. The genes encoding the ECM components *trol* (Perlecan), *vkg* and *col4a1* (CollagenIV), *dally* (heparansulfate proteoglycan), *Tigrin* and *SPARC* are strongly expressed in ensheathing glia (arrowheads). The genes encoding the different Laminin subunits (*LanA*, *LanB1*, *LanB2*) are weakly expressed by glia. Source data are from the Aerts laboratory [11].

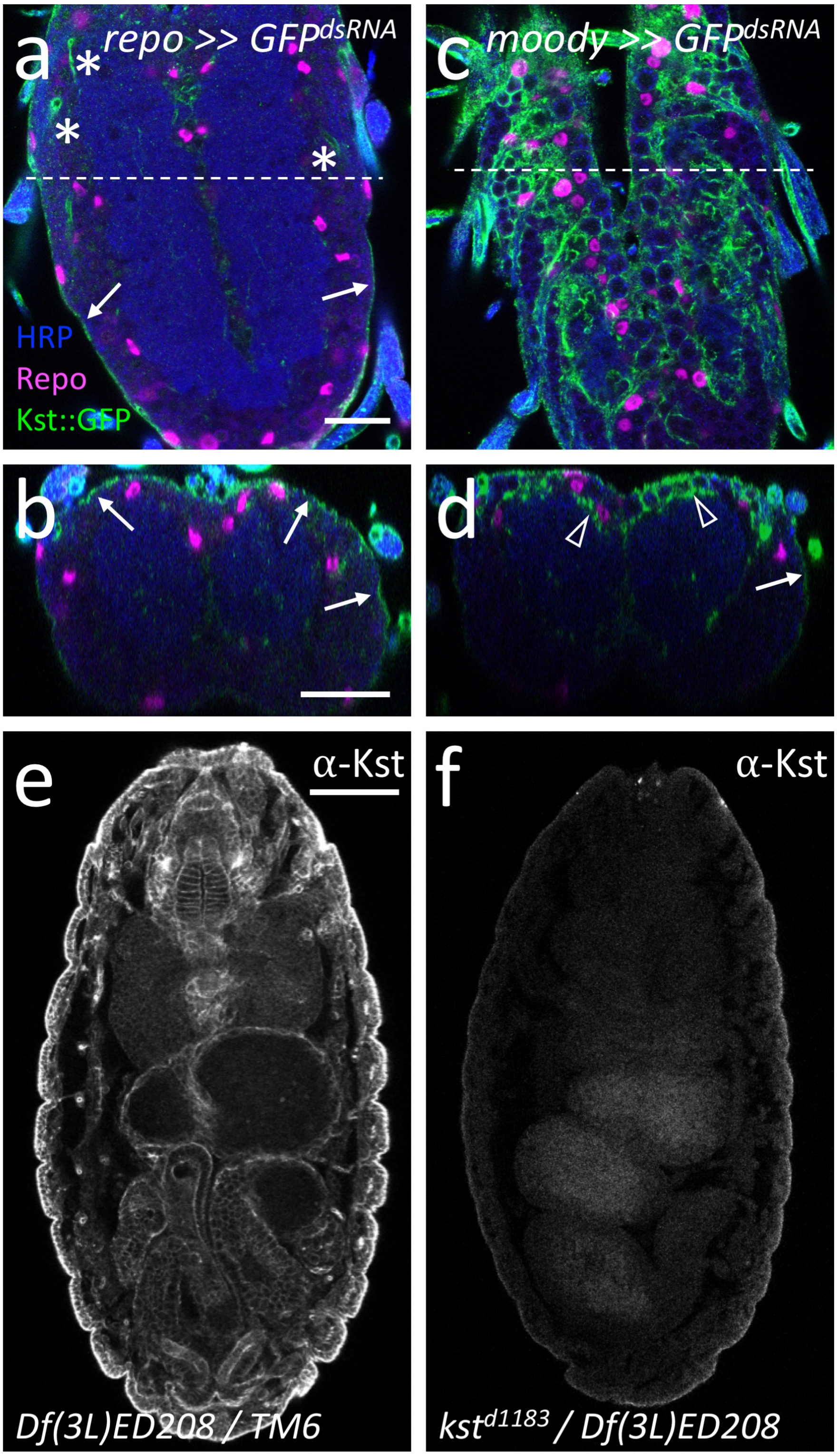

Supplementary Fig. S7 Karst expression

a-d Silencing of Karst^{GFP} expression in larval brains. **a** Single focal plane of a CNS with the genotype [*repo-Gal4, UAS-GFP^{dsRNA}, kst$^{MiMIC::GFP}$*]. The position of trachea is indicated (asterisks). The dashed line shows the position of the orthogonal section shown in (**b**). The arrows point to unspecific binding of the anti-GFP antibody to the outer surface of the CNS. **c,d** Single focal plane of a CNS with the genotype [*moody-Gal4, UAS-GFP^{dsRNA}, kst$^{MiMIC::GFP}$*]. Note, that GFP expression is still found around the neuropil (arrowheads). **e** Stage 16 control embryo and (**f**) *kst* deficient embryo stained for Karst localization. To obtain specific antibodies, rabbits were immunized using a short peptide (^{3622}LADERRRAEKQHEHRQN^{3639}) shared by all ß$_H$-Spectrin proteins. The purified antiserum was used to stain control and *karst* null mutant embryos as indicated. Representative images are shown. Scale bars are 50 µm.

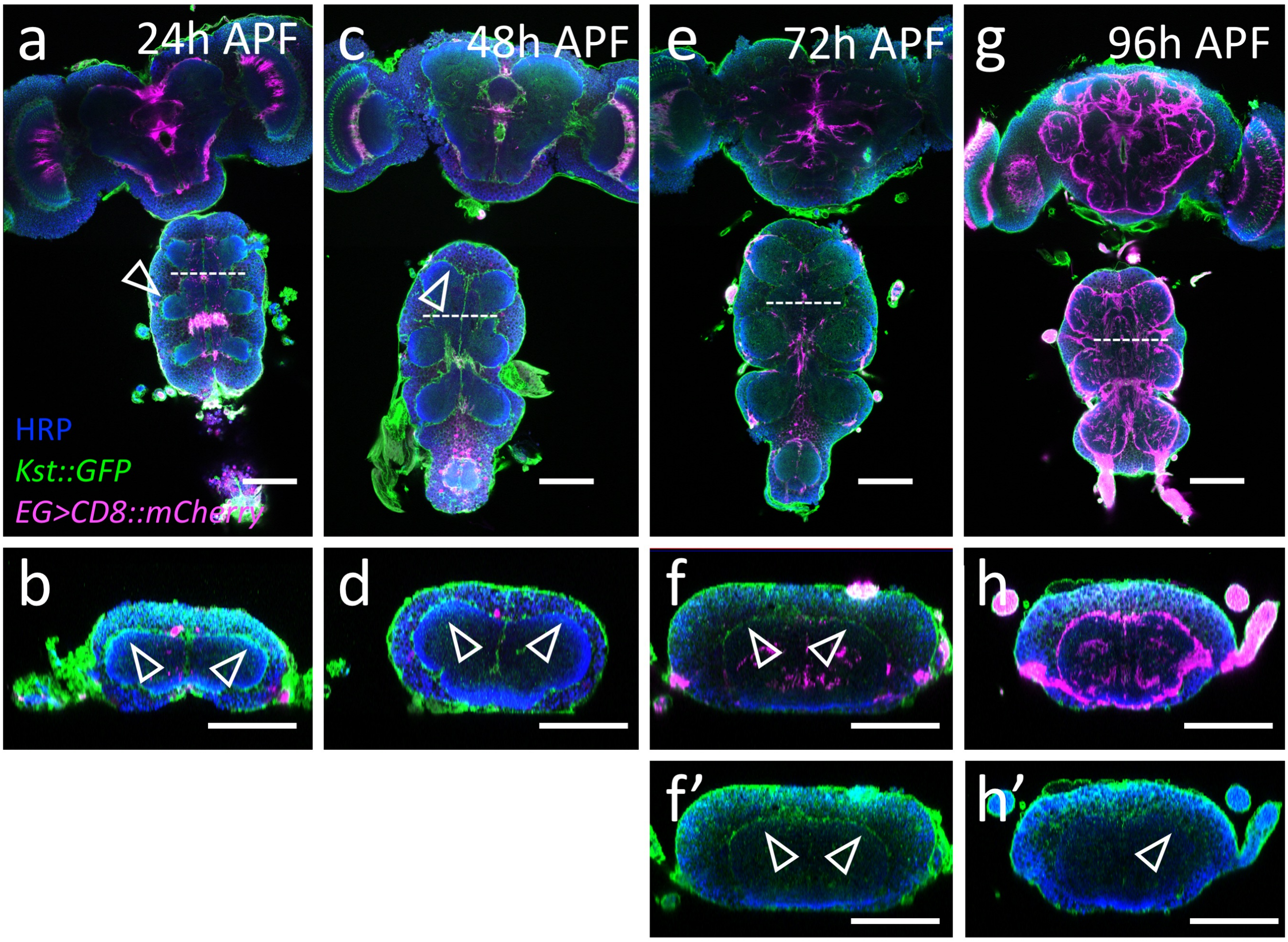

Supplementary Fig. S8 Karst expression in ensheathing glia declines during pupal development
Differentially aged pupal brains with the hours after puparium formation (APF) indicated were stained for Karst::GFP (green), CD8::mCherry expression driven by *83E12-Gal4* (magenta) and HRP (blue) to label all neuronal membranes. The positions of the orthogonal sections are indicated by dashed lines. **a-f** *Karst::GFP* expression can be detected up to 72h APF. **g,h** In 96 h APF old pupae, ensheathing glia reorganize and Karst expression disappears. Representative images are shown. Scale bar is 100 µm.

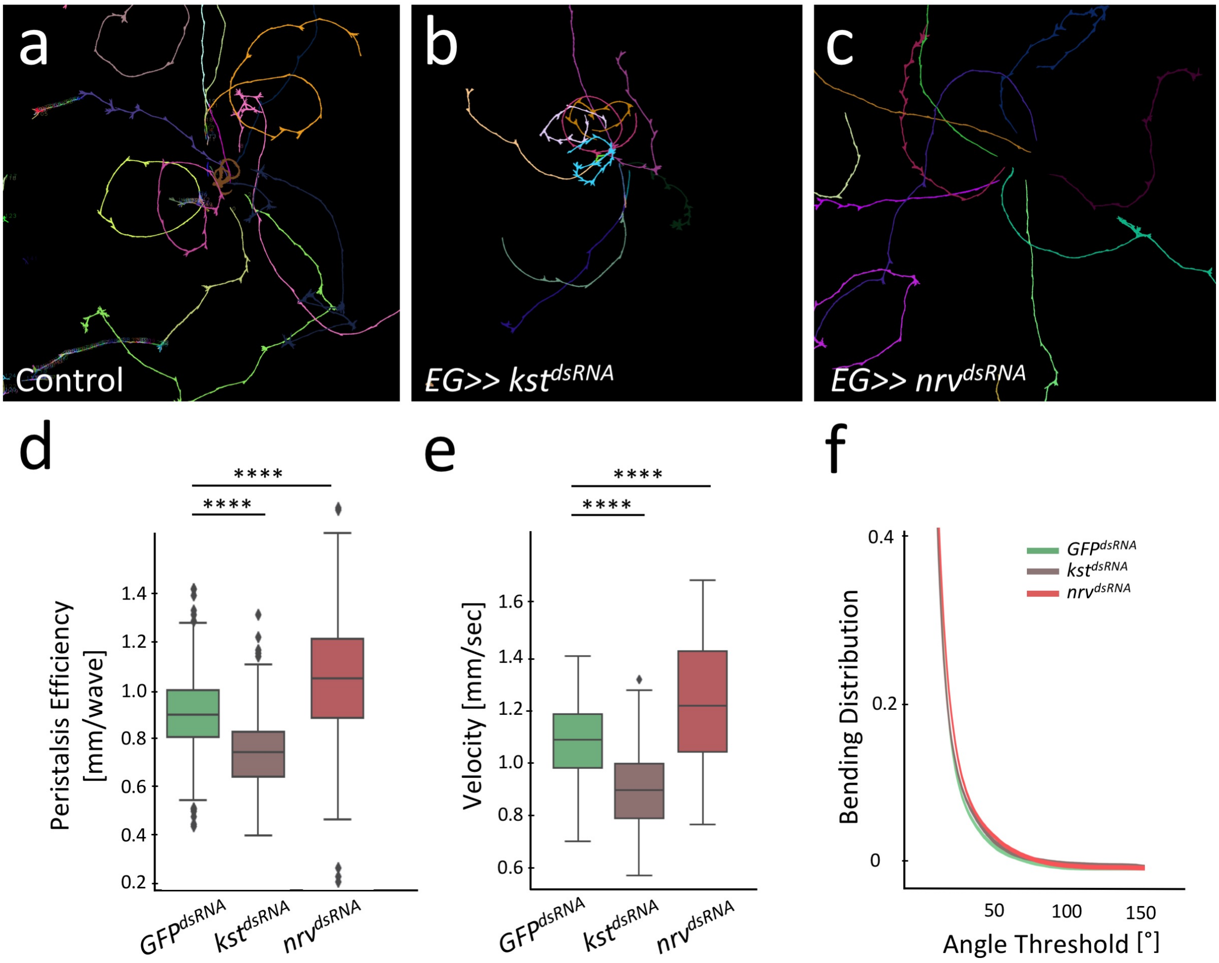

Supplementary Fig. S9 Opposite effects of *karst* and *nrv2* on larval locomotion

a-c Representative larval locomotion tracts of the genotypes indicated. **d** Quantification of peristalsis efficiency. Box plots show median (line), boxes represent the first and third percentiles, whiskers show standard deviation, diamonds indicate outliers. Note, that knockdown of *nrv2* specifically in ensheathing glia results in an increase in the peristalsis efficiency [*83E12-Gal4^{AD}, repo-Gal4^{DBD}, nrv2^{dsRNA}*], whereas knockdown of *karst* results in a decrease [*83E12-Gal4^{AD}, repo-Gal4^{DBD}, kst^{dsRNA}*]. **e** Quantification of crawling velocity. Knockdown *nrv2* leads to an increase in the crawling velocity, whereas knockdown of *karst* results in a decrease. **f** Quantification of bending distribution. Quantification Wilcoxon rank-sum test, n=50. Peristalsis Efficiency [mm/wave]: *GFP^{dsRNA}* – *kst^{dsRNA}*: **** p = 3.22E-30; *GFP^{dsRNA}* – *nrv^{dsRNA}*: **** p = 1.20E-17; velocity [mm/sec]: *GFP^{dsRNA}* - *kst^{dsRNA}*: **** p = 3.81E-11;; *GFP^{dsRNA}* – *nrv^{dsRNA}*: **** p = 8.49E-05.

Supplementary movie 1

Supplementary movie 2
