# Supplementary material for: Drosophila ßHeavy-Spectrin is required in polarized ensheathing glia that form a diffusion-barrier around the neuropil
Source: Nat Commun. 2021 Nov 4;12:6357. doi: 10.1038/s41467-021-26462-x (PMC8569210; doi:10.1038/s41467-021-26462-x)
Supplement: Supplementary file 4 — Description of Additional Supplementary Files [file 41467_2021_26462_MOESM4_ESM.pdf]

Title: Supplementary movie 1

Description: Dye penetration in control third instar larvae. 10 kDa Texas-Red conjugated dextran was injected into the neuropil of one brain hemisphere of a control third instar larva with the genotype [*83E12-Gal4, UAS-CD8::GFP*] about 5 minutes after preparation. Diffusion of the dye to the contralateral side was imaged for 25 minutes. A time stamp in minutes is indicated. The first 10 minutes were quantified (see Figure 5c).

Title: Supplementary movie 2

Description: Dye penetration in ensheathing glia ablated third instar larvae. 10 kDa Texas-Red conjugated dextran was injected into the neuropil of one brain hemisphere of a control third instar larva with the genotype [*UAS-rpr; 83E12-Gal4*^{AD} *repo-Gal4*^{DBD}*, UAS-hid*] about 5 minutes after preparation. Diffusion of the dye to the contralateral side was imaged for 20 minutes. A time stamp in minutes is indicated. The first 10 minutes were quantified (see Figure 5c).
