# Supplementary material for: Drosophila ßHeavy-Spectrin is required in polarized ensheathing glia that form a diffusion-barrier around the neuropil
Source: Nat Commun. 2021 Nov 4;12:6357. doi: 10.1038/s41467-021-26462-x (PMC8569210; doi:10.1038/s41467-021-26462-x)
Supplement: Supplementary file 5 — Reporting Summary [file 41467_2021_26462_MOESM5_ESM.pdf]

nature portfolio

Corresponding author(s): Christian Klämbt

Last updated by author(s): 9/13/2021

Reporting Summary

Nature Portfolio wishes to improve the reproducibility of the work that we publish. This form provides structure for consistency and transparency in reporting. For further information on Nature Portfolio policies, see our Editorial Policies and the Editorial Policy Checklist.

Statistics

For all statistical analyses, confirm that the following items are present in the figure legend, table legend, main text, or Methods section.

n/a	Confirmed	
☐	☒	The exact sample size (*n*) for each experimental group/condition, given as a discrete number and unit of measurement
☐	☒	A statement on whether measurements were taken from distinct samples or whether the same sample was measured repeatedly
☐	☒	The statistical test(s) used AND whether they are one- or two-sided<br>*Only common tests should be described solely by name; describe more complex techniques in the Methods section.*
☒	☐	A description of all covariates tested
☒	☐	A description of any assumptions or corrections, such as tests of normality and adjustment for multiple comparisons
☐	☒	A full description of the statistical parameters including central tendency (e.g. means) or other basic estimates (e.g. regression coefficient) AND variation (e.g. standard deviation) or associated estimates of uncertainty (e.g. confidence intervals)
☐	☒	For null hypothesis testing, the test statistic (e.g. *F*, *t*, *r*) with confidence intervals, effect sizes, degrees of freedom and *P* value noted<br>*Give P values as exact values whenever suitable.*
☒	☐	For Bayesian analysis, information on the choice of priors and Markov chain Monte Carlo settings
☒	☐	For hierarchical and complex designs, identification of the appropriate level for tests and full reporting of outcomes
☒	☐	Estimates of effect sizes (e.g. Cohen's *d*, Pearson's *r*), indicating how they were calculated

Our web collection on statistics for biologists contains articles on many of the points above.

Software and code

Policy information about availability of computer code

Data collection	FIMTrack v3.1.28.5, Zeiss imager ZEN 2.3, Leica LasX 3.5.7.23225
Data analysis	FIMAnalytics v0.1.1.2, Excel15.28, Fiji, version 1.52b; Prism 6.0, CATMAID, Imaris 8.4.1, Adobe Photoshop CS6

For manuscripts utilizing custom algorithms or software that are central to the research but not yet described in published literature, software must be made available to editors and reviewers. We strongly encourage code deposition in a community repository (e.g. GitHub). See the Nature Portfolio guidelines for submitting code & software for further information.

Data

Policy information about availability of data

All manuscripts must include a data availability statement. This statement should provide the following information, where applicable:

- Accession codes, unique identifiers, or web links for publicly available datasets
- A description of any restrictions on data availability
- For clinical datasets or third party data, please ensure that the statement adheres to our policy

The fly stocks and datasets generated during and analyzed during the current study are available from the corresponding author on reasonable request.

Field-specific reporting

Please select the one below that is the best fit for your research. If you are not sure, read the appropriate sections before making your selection.

- [x] Life sciences
- [ ] Behavioural & social sciences
- [ ] Ecological, evolutionary & environmental sciences

For a reference copy of the document with all sections, see nature.com/documents/nr-reporting-summary-flat.pdf

Life sciences study design

All studies must disclose on these points even when the disclosure is negative.

Sample size	The sample size was selected based on previous experience and on the current standard in the field. For histochemical analyses more than 10 animals originating from at least three crosses were analyzed. For behavioral studies we analyzed more than 45 animals from at least three independent crosses for 180 seconds generating 1800 frames per animal. Generally highly significant p-values were obtained in statistical tests. The TEM volumes generated at Janelia farms were analyzed (one per developmental stage).
Data exclusions	No data exclusion for behavioral experiments.
Replication	All attemps to replicate the data were successful.
Randomization	Larvae were grouped according to their genotype, within a given genotype animals of comparable size were selected.
Blinding	All behavioral analyses were done by FIManalytics from movies taken from behaving larvae. EM analysis was performed non-blinded, since there is only one dataset (wild type larva), blinding is not possible. LSM analysis. The different genotypes analyzed were genetically distinct and only defined groups of mutants were studied. Blinding is thus not necessary. We did however, performed blinded analysis of the images to ensure that the phenotypes are all correctly described, e.g. Nicole Pogodalla took the images and quantified them according to the pre-defined criteria. I subsequently looked at all images blinded and separated them according to their phenotype - which in all cases matched the expected genotypes.

Reporting for specific materials, systems and methods

We require information from authors about some types of materials, experimental systems and methods used in many studies. Here, indicate whether each material, system or method listed is relevant to your study. If you are not sure if a list item applies to your research, read the appropriate section before selecting a response.

Materials & experimental systems

n/a	Involved in the study	
[ ]	[x]	Antibodies
[x]	[ ]	Eukaryotic cell lines
[x]	[ ]	Palaeontology and archaeology
[ ]	[x]	Animals and other organisms
[x]	[ ]	Human research participants
[x]	[ ]	Clinical data
[x]	[ ]	Dual use research of concern

Methods

n/a	Involved in the study	
[x]	[ ]	ChIP-seq
[x]	[ ]	Flow cytometry
[x]	[ ]	MRI-based neuroimaging

Antibodies

Antibodies used	anti-Repo (8D12; Developmental Studies Hybridoma bank); anti-mCherry (Cat# M11217); anti-V5 (Cat# R960-25) and DAPI (Cat# D1306) (all from Invitrogen™); anti-Flag (Cat# SAB4200071, Sigma-Aldrich); anti-Ollas (Cat# MAB7372, Abnova), anti-HA (Cat# 9015116, BioLegend); anti-GFP (Cat# A-6455, Life Technologies); Anti-phospho-histone H3 (phospho S28) (Cat# ab10543, Abcam); anti-HRP-Alexa Flour 647 conjugated (Cat# 123-005-021, Dianova); anti-Nazgul was a gift from B. Altenhein, Cologne. Rabbit anti-Rumpel was a gift of K. Yildirim, Münster. A guinea Pig anti-serum generated against a GST -EAAT2 fusion protein (Peco et al., 2016) was a gift of D. van Meyel. A C-terminally located peptide (3622LADERRRAEKQHEHRQN3639) shared by all ßH-Spectrin (Kars) proteins was used to immunize rabbits (Pineda, Berlin).
Validation	All antibodies were validated by expression analysis. Antibodies against tags (V5, Flag, HA, Ollas, dsRed, GFP, Cherry) did not stain animals lacking expression of the tag, but did stain when we expressed respectively tagged proteins.

Animals and other organisms

Policy information about studies involving animals; ARRIVE guidelines recommended for reporting animal research

Laboratory animals	Study involved Drosophila melanogaster. Equal numbers of female and male five days old third instar larvae were analyzed. The following strains were used: 83E12-Gal4 (BDSC#40363), 83E12-LexA (BDSC #54288), P{y[+t7.7] w[+mC]=13XLexAop2-mCD8::GFP}

attP2 (BDSC#32203), MCFO-2: pBPhsFlp2::PEST;; HA_V5_FLAG_OLLAS (BDSC#64086), stringdsRNA (#1395R-2), P{w[+mC]=UAS-htl.lambda\cI.M}40-22-2 (108191), P{w[+mC]=UAS-Lam.GFP}3-3 (BDSC#7376), P{w[+mC]=UAS-mCD8::GFP.L}LL6 (BDSC#5130), P{UAS-mCD8.ChRFP}2 (BDSC#27391), P{w[+mC]=UAS-rpr.C}27 (BDSC#5823), P{w[+mC]=UAS-hid.Z}2/CyO (BDSC#65403), P{4C.UAS-PLCδ-PH-mCherry} (BDSC#51658), PBac{806.LOX-SVS-2}ifCPTI004152 (#4152), Mi{PT-GFSTF.0}trolMI04580-GFSTF.0 (BDSC#60214), Mi{PT-GFSTF.1}kstMI03134-GFSTF.1 (BDSC#60193), Mi{MIC}kstMI03134 (BDSC#36203), Mi{MIC}kstMI13613 (BDSC#59172), P{XP}kstd11183 (BDSC#19345), kstP{EPgy2}EY01010 (BDSC#15488), kstdsRNA (BDSC#50536), Df(3L)ED208 (BDSC#8059), , Mi{PT-GFSTF.1}nrv2MI03354-GFSTF.1 (BDSC#59407), nrv2dsRNA (VDRC#2260), P{w[+mC]=lexAop-rCD2.RFP}2; P{w[+mC]=UAS-CD4-spGFP1-10}3, P{w[+mC]=lexAop-CD4-spGFP11}3 (BDSC#58755), 55B12-Gal4 (BDSC#39103), VGlut[OK371]-Gal4 (BDSC#26160), GFPdsRNA (BDSC#9331).
The following strains were generated in this study
83E12-Gal4AD, repo -Gal4DBD, dally::GFP, Mi{Trojan-GAL4.0} kstMI03134

Wild animals	No wild animals were used in this study
Field-collected samples	No field-collected animals were used in this study
Ethics oversight	According to German and European law, work with Drosophila melanogaster does not require approval by the local Ethics committee.

Note that full information on the approval of the study protocol must also be provided in the manuscript.
